# Including non-additive genetic effects in Bayesian methods for the prediction of genetic values based on genome-wide markers

**DOI:** 10.1186/1471-2156-12-74

**Published:** 2011-08-25

**Authors:** Dörte Wittenburg, Nina Melzer, Norbert Reinsch

**Affiliations:** 1 Research Unit Genetics and Biometry, Leibniz Institute for Farm Animal Biology (FBN), Wilhelm-Stahl-Allee 2, 18196 Dummerstorf, Germany

## Abstract

**Background:**

Molecular marker information is a common source to draw inferences about the relationship between genetic and phenotypic variation. Genetic effects are often modelled as additively acting marker allele effects. The true mode of biological action can, of course, be different from this plain assumption. One possibility to better understand the genetic architecture of complex traits is to include intra-locus (dominance) and inter-locus (epistasis) interaction of alleles as well as the additive genetic effects when fitting a model to a trait. Several Bayesian MCMC approaches exist for the genome-wide estimation of genetic effects with high accuracy of genetic value prediction. Including pairwise interaction for thousands of loci would probably go beyond the scope of such a sampling algorithm because then millions of effects are to be estimated simultaneously leading to months of computation time. Alternative solving strategies are required when epistasis is studied.

**Methods:**

We extended a fast Bayesian method (fBayesB), which was previously proposed for a purely additive model, to include non-additive effects. The fBayesB approach was used to estimate genetic effects on the basis of simulated datasets. Different scenarios were simulated to study the loss of accuracy of prediction, if epistatic effects were not simulated but modelled and vice versa.

**Results:**

If 23 QTL were simulated to cause additive and dominance effects, both fBayesB and a conventional MCMC sampler BayesB yielded similar results in terms of accuracy of genetic value prediction and bias of variance component estimation based on a model including additive and dominance effects. Applying fBayesB to data with epistasis, accuracy could be improved by 5% when all pairwise interactions were modelled as well. The accuracy decreased more than 20% if genetic variation was spread over 230 QTL. In this scenario, accuracy based on modelling only additive and dominance effects was generally superior to that of the complex model including epistatic effects.

**Conclusions:**

This simulation study showed that the fBayesB approach is convenient for genetic value prediction. Jointly estimating additive and non-additive effects (especially dominance) has reasonable impact on the accuracy of prediction and the proportion of genetic variation assigned to the additive genetic source.

## 1 Background

Molecular marker information is commonly used to draw inferences about the relationship between genetic and phenotypic variation in various species, e.g. humans [1], dairy cattle [2] or mice [3]. Assuming linkage disequilibrium (LD) between quantitative trait loci (QTL) and markers, genetic effects can be estimated and explained as QTL effects captured by the neighbouring markers. If breeding values are the focal point, genetic effects are typically modelled as additively acting marker allele effects (e.g. [4,5]). The mode of biological action can, of course, be different from the assumption of pure additivity. One possibility to better understand the genetic architecture of complex traits is to include intra-locus (dominance) and inter-locus (epistasis) interaction of alleles when fitting a model to a trait. The importance of non-additive effects for genetic variation has recently been investigated. Knowledge about non-additive effects is essential to benefit, for example, from heterosis effects [6], especially for cross-breeding schemes (poultry, plants etc.). In general, it can be expected that the prediction of the genetic value, in particular its additive part, is improved if non-additive effects are additionally modelled. For instance, Lee *et al. *[7] reported that the accuracy of prediction increased considerably when dominance effects were included compared to a purely additive genetic model when the phenotypes coat colour (+17% accuracy) or the percentage of CD8^+ ^cells (+2% accuracy) were studied in mice. Added epistasis did not, however, contribute to the accuracy in this case. In an example with recombinant inbred lines of soybean [8], the accuracy of prediction was more than doubled under the epistatic model. Even though non-additive effects may occur on the level of gene action, most of the genetic variation might be assigned to additive effects when genes are at an extreme frequency [9]. The extent to which, for example, epistasis is involved in regulating complex traits is hardly known, but knowledge about it can be used to infer biological mechanisms and to reconstruct biological pathways [10]. In one of the first studies concerning non-additive influence on growth differences in chickens, Carlborg *et al. *[11] estimated that 10% of genetic variation in early growth (trait Gr18) was due to dominance and even 70% due to epistasis. This example showed the importance of interacting loci, though one may suppose an overestimation of the epistatic effects, a phenomenon already known as the Beavis effect [12] for single loci. Since this experiment was based on a cross of extremely different lines, further investigations are required to find evidence for interacting genes in purebreds.

Different approaches are available to model additive and non-additive genetic effects. Under the aspect of QTL detection, a genome scan can be carried out to uncover genetic effects using, for example, a variance component method [13,14]. If additive and non-additive effects are to be modelled simultaneously over the whole genome, we have to be aware of "*p *bigger than *n*" problems, meaning there are more parameters than there are observations. To cope with the all-in-one situation, Xu presented a Bayesian approach [15], which parallels the idea of BayesA [4], and an empirical Bayes method [16] both enabling the genome-wide estimation of additive and non-additive marker effects. The Bayesian methods commonly used for the estimation of additive effects apply Markov chain Monte Carlo (MCMC) simulations which require a lot of computing time, but they convince in terms of accuracy in predicting genetic values. In particular, the BayesB approach [4] is superior to other methods, for instance ridge regression and partial least squares [17-19]. The MCMC sampling methods may collapse under high marker density if further non-additive effects are included. As an alternative, an approximate Bayesian approach is available which applies the analytically derived posterior density for a marker effect rather than samples thereof [20]. This approach (called fBayesB) was shown to be slightly less accurate, because in an iterative procedure only a single marker effect is studied at a time while the vector of phenotypes is corrected for all other previously estimated effects. The fBayesB strategy is much faster than the conventional Bayesian methods using MCMC. This solving approach offers the possibility to additionally account for genome-wide interacting effects and to estimate them with reasonable computational effort.

The objective of this study is to explore the impact of non-additive effects on the prediction of genetic values in a livestock population. An improved estimation of additive effects and a better prediction of genetic values is intended, when additive and non-additive effects are jointly involved in fitting a model to a trait. Since methods that aim to estimate non-additive effects in arbitrary populations are just emerging, it is especially important to validate such approaches with simulations. Therefore, with this study, we pursue methodological aspects, thereby assembling facts that help to interpret results obtained with practical data in future work. We consider additive, dominance and pairwise epistatic effects captured by biallelic markers spread over the whole genome. The details of statistical modelling are presented in the first part of the paper. We extend the fast Bayesian method (fBayesB), which was developed under pure additivity [20], to include non-additive effects. fBayesB is used to estimate the genetic effects on the basis of simulated datasets which resemble a dairy cattle population. Different scenarios are simulated to study the loss of accuracy of prediction if epistatic effects are not simulated but modelled and vice versa. In the second part, we summarise the results of analysing the simulated data. The amount of genetic variation assigned to each kind of genetic effect after genome-wide estimation of marker effects is determined. To briefly show how the approach behaves in practice, we also apply fBayesB to a real data example. In the third part, we outline some constraints of estimating non-additive effects via the fBayesB approach and discuss other solving strategies.

## 2 Methods

### 2.1 Statistical model

For the statistical analysis of genetic effects in a Bayesian framework, a hierarchical model is constructed similar to that of Meuwissen *et al. *[20]. Bold symbols are used for vectors and matrices. At first, only main genetic effects (i.e. additive and dominance effects) are included. In total *m *loci are studied on the genome. The vector of phenotypes ***y ***= (*y*_1_, ..., *y_n_*)' is modelled as

y=1μ+Xa+Dd+e.

This model is set up in the way of an F_∞ _model [21]. Let *μ *be a population mean and **1 **a vector of ones. The ***X ***and ***D ***are design matrices for allele substitution effects ***a ***= (*a*_1_, ..., *a_m_*)' and dominance effects ***d ***= (*d*_1_, ..., *d_m_*)', respectively. The entries of the design matrices are random variables which are realised depending on the observed marker genotypes (denoted as 11, 12, 22). For a homozygous genotype at locus *j *∈ {1, ..., *m*} of animal *i *∈ {1, ..., *n*}, *X_i,j _*= ± 1 and *D_i,j _*= 0; the positive effect is assigned to the more frequent allele. For a heterozygous genotype, *X_i,j _*= 0 and *D_i,j _*= 1.

This work relies on two assumptions. Firstly, linkage equilibrium (LE) between the different markers is assumed. Then genotypic effects at different loci are independently distributed and the estimation strategy does not depend on the order of markers. Secondly, in order to avoid the estimation of covariance components at intra-locus investigations, the additive genetic value and the dominance genetic value are assumed uncorrelated at each locus, i.e. Cov(*X_i,j_a_j_, D_i,j_d_j_*) = 0 ∀*i,j*. This assumption can be fulfilled by re-parametrising coefficients coding for the marker genotypes in advance. We apply the method of Álvarez-Castro & Carlborg [22] to obtain an orthogonal decomposition of genetic values. This method involves the genotype frequencies *p*_11,*j*_, *p*_12,*j*_, *p*_22,*j *_at each locus *j *and does not necessarily depend on Hardy-Weinberg equilibrium (HWE). The method is related to Cockerham's model [23] given HWE. In an F_∞ _model, the genotypic effects ***G**_j _*= (*G*_11,*j*_, *G*_12,*j*_, *G*_22,*j*_)' can be written as

(1) $$G_{j}=S\left(\begin{array}{c} \mu \\ a_{j}\\ d_{j} \end{array}\right),\;S=\left(\begin{array}{c} 1\;-1\;0\\ 1\;0\;1\\ 1\;1\;0 \end{array}\right).$$

The second and third column of ***S ***represent the possible realisations in ***X ***and ***D***, respectively. The genotypic values can also be obtained in terms of an additive effect *g_a, j _*and dominance effect *g_d, j _*on the orthogonal scale by

(2) $$G_{j}=S_{A,j}\left(\begin{array}{c} \mu^{*}\\ g_{a,j}\\ g_{d,j} \end{array}\right)\text{with}$$

$$S_{A,j}=\left(\begin{array}{c} 1\;-p_{11,j}-2p_{22,j}\;-\frac{2p_{12,j}p_{22,j}}{v}\\ 1\;1-p_{11,j}-2p_{22,j}\;-\frac{4p_{11,j}p_{22,j}}{v}\\ 1\;2-p_{11,j}-2p_{22,j}\;-\frac{2p_{12,j}p_{22,j}}{v} \end{array}\right),$$

where *v *= *p*_11,*j *_+ *p*_22,*j *_- (*p*_11,*j *_- *p*_22,*j*_)^2^. Since the representations (1) and (2) are equivalent [22], the F_∞ _model can be translated into

(M1) $$y=1\mu^{*}+X_{a}g_{a}+X_{d}g_{d}+e,$$

where the design matrices ***X**_a _*and ***X**_d _*contain the corresponding entries of ***S**_A,j _*(*j *= 1, ..., *m*) and relate to the additive and dominance effects on the orthogonal scale, respectively.

To obtain numerical stability in later calculations, coefficients of the main genetic effects are additionally standardised. Let *p_j _*denote the (estimated) allele frequency at locus *j*. One possibility is to divide the columns in ***X**_a _*and ***X**_d _*by the standard deviation of the random variable coding the marker genotype for the additive or dominance effects, respectively,

(3) $$\begin{array}{c} X_{a,j}\mapsto \frac{X_{a,j}}{\sqrt{2p_{j}\left(1-p_{j}\right)}}\;\text{and}\\ X_{d,j}\;\mapsto \;\frac{X_{d,j}}{2p_{j}\left(1-p_{j}\right)}. \end{array}$$

Now the hierarchical structure of M1 can be characterised by the following prior distributions

$$\begin{array}{c} e_{i}\;\sim N\left(0,\sigma_{e}^{2}\right),\;i=1,\;\ldots ,\;n,\\ g_{s,j}\sim \;L^{*}\left(\gamma_{s},\lambda_{s}\right),\;\;s\;\in \left\{a,\;d\right\},\;j=1,\;\ldots ,\;m. \end{array}$$

L*(*γ_s_*, *λ_s_*) denotes a mixture of a Laplace distribution with zero expectation and the point mass at zero. The mixing probability is *γ_s_*, then Pr(*g_s,j _*= 0) = 1 - *γ_s_*. Furthermore, $\text{Var}(g_{s,j})=\gamma_{s}\frac{2}{\lambda_{s}^{2}}$, where *λ_s _*denotes a measure of uncertainty about the effects of the genetic variation source *s*. The hyper-parameters *γ_s _*and *λ_s _*are specified for each source, either additive (*s *= *a*) or dominance (*s *= *d*).

In a second step, the pairwise epistatic effects are modelled. The genetic effect caused by an interaction between locus *j *and *k *is denoted as *g_s,j,k _*with *s *∈ {*aa, ad, da, dd*}. The effect is considered additive × additive (*aa*), only if the individual *i *is homozygous at the loci *j *and *k*. It is considered additive × dominance (*ad*), when is appears at a homozygous locus *j *and a heterozygous locus *k *(*j *<*k*) and dominance × additive (*da*) for the reverse case. The dominance × dominance effect (*dd*) appears between heterozygous loci. Using the already orthogonalised columns in ***X**_a _*and ***X**_d_*, M1 can be extended to include epistatic effects in a way similar to Kao & Zeng [21]. Let *s *∈ {*aa, ad, da, dd*},

(M2) $$y=1\mu^{*}+X_{a}g_{a}+X_{d}g_{d}+\underset{s}{\sum }X_{s}g_{s}+e.$$

As an example, ***X**_aa,j,k _*= ***X**_a,j _· **X**_a,k_*, where the symbol · denotes the element-wise multiplication of column *j *and *k *of ***X**_a_*. Furthermore, ***X**_ad,j,k _*= ***X**_a,j _*· ***X**_d,k _*is calculated to obtain the coefficients for the effect *g_ad,j,k_*. This way, in total four times $\frac{m\left(m-1\right)}{2}$ epistatic effects are modelled. The prior remains the same as for M1, but we assume that the probability of having non-zero epistatic effects is smaller than the *γ_s _*for main effects.

### 2.2 Parameter estimation

The essence of the fBayesB approach is the iterative conditional expectation (ICE) algorithm, which is described in detail by Meuwissen *et al. *[20]. We only describe the steps which were adapted under the influence of non-additive genetics. Initially, to get rid of the population mean μ*, we shift the observed phenotypic values by the estimated mean value, thus $y\mapsto y-1\bar{y}$. The vector of genetic effects ***g**_s _*has the length *m_s_*, where *m_s _*= *m *for *s *∈ {*a, d*} and $m_{s}=\frac{m\left(m-1\right)}{2}$ for *s *∈ {*aa, ad, da, dd*}. In case of epistasis, the elements are stored in a vector according to a vectorised upper triangular matrix, where only elements above the diagonal are taken, i.e.

$$g_{s}\;=\;{\left(g_{s,1,2},\;g_{s,1,3},\;\ldots ,\;g_{s,1,m},\;g_{s,2,3},g_{s,2,4},\;\ldots ,\;g_{s,2,m},\;\ldots ,\;g_{s,m-1,m},\;\right)}^{\prime }.$$

We carry out *k *= 1, 2, ..., *k*_max _iterations and process the genetic effects in the order *s *= *a*, *d *based on M1 or *s *= *a, d, aa, ad, da, dd *based on M2. The genetic effect with index *j *= 1, ..., *m_s _*is estimated as the posterior expectation

$${ĝ}_{s,j}^{\left(k\right)}\;=\;E\left(g_{s,j}|y=y_{-j}^{\left(k\right)}\right),$$

where $y_{-j}^{\left(k\right)}$ denotes the vector of observed phenotypes corrected for all estimated effects except the *j*-th effect in iteration round *k*.

Set $Y_{j}={\left(X_{s,j}^{\prime }X_{s,j}\right)}^{-1}X_{s,j}^{\prime }y_{-j}^{\left(k\right)}$ and $\sigma_{j}^{2}={\left(X_{s,j}^{\prime }X_{s,j}\right)}^{-1}\sigma_{e}^{2}$. For convenience we denote $Y_{j}^{\pm }=Y_{j}\pm \lambda_{s}\sigma_{j}^{2}.$

Now the conditional expectation was determined analytically in Meuwissen *et al. *[20] as

$$\begin{array}{c} E\left(g_{s,j}|y=y_{-j}^{\left(k\right)}\right)\\ =\frac{T_{1}\Theta_{U}\left(0;Y_{j}^{-},\sigma_{j}^{2}\right)+T_{2}\Theta_{L}\left(0;Y_{j}^{+},\sigma_{j}^{2}\right)}{T_{1}+T_{2}+T_{3}} \end{array}$$

With

T1=exp(-λsYj)(1-Φ(0;Yj-,σj2)),T2=exp(λsYj)Φ(0;Yj+,σj2),T3=2(1-γs)γsλs exp(-12λs2σj2)ϕ(Yj;0,σj2).

The Θ*_U _*(0; *μ*, *σ*^2^) and Θ*_L_*(0; *μ*, *σ*^2^) are the expected value of an upper and lower truncated normal distribution *N*(*μ*, *σ*^2^), respectively, with truncation point zero. The Φ(*x*; *μ*, *σ*^2^) denotes the normal distribution function evaluated at some point *x *and *ϕ*(*x*; *μ*, *σ*^2^) is the normal density function.

We introduce a slight modification to fBayesB as we update the estimated residual variance components in each iteration *k *by the residual sum of squares

$${\hat{\sigma }}_{e}^{2\left(k\right)}\;=\;\frac{1}{n-1}{∥y-\underset{s}{\sum }X_{s}{\hat{g}}_{s}^{\left(k\right)}∥}^{2}.$$

Then $\sigma_{e}^{2}$ is substituted by ${\hat{\sigma }}_{e}^{2\left(k\right)}$ in the calculation of the conditional expectation. The steps above are carried out for all indices *j *within each source *s *of genetic variation. We continue until the vector of estimates ${\hat{g}}^{\left(k\right)}={\left({\hat{g}}_{a}^{{\left(k\right)}^{\prime }},{\hat{g}}_{d}^{{\left(k\right)}^{\prime }}{\hat{g}}_{aa}^{{\left(k\right)}^{\prime }},{\hat{g}}_{ad}^{{\left(k\right)}^{\prime }},{\hat{g}}_{da}^{{\left(k\right)}^{\prime }},{\hat{g}}_{dd}^{{\left(k\right)}^{\prime }}\right)}^{\prime }$ fulfils the convergence criterion

$$\frac{||{\hat{g}}^{\left(k\right)}-{\hat{g}}^{\left(k-1\right)}|{|}^{2}}{||{\hat{g}}^{\left(k\right)}|{|}^{2}}\leq L,$$

otherwise the iterations stop at *k *= *k*_max_. The (direct) genetic value *DGV_i _*of individual *i *is obtained as the genome-wide sum over all genotypic values and over the different sources, i.e.

$$DGV_{i}\;=\;\underset{s}{\sum }X_{s}{\hat{g}}_{s}.$$

Eventually, as a consequence of the standardisation, the genetic variance components are estimated as ${\hat{\sigma }}_{s}^{2}=\overset{m_{s}}{\underset{j=1}{\sum }}{ĝ}_{s,j}^{2}$for each genetic variation source *s*. Note that this formula yields an approximation under LD because the covariance components Cov$\left(X_{s,i,j}{ĝ}_{s,j},X_{s,i^{\prime },j^{\prime }}{ĝ}_{s,j^{\prime }}\right)$ of potentially linked loci *j *and *j*' are absent. Under the given re-parametrisation, the covariance Cov(*X*_*s,i,j*_, *X*_*s,i',j'*_) between genotype coefficients is not necessarily positive and the signs of the corresponding effects are not known. Therefore, it cannot be stated whether over- or underestimation of genetic variance components is expected. We briefly examine the impact of missing linkage information in our simulations.

The suitability of the statistical models M1 and M2 are compared among the different simulated scenarios in terms of accuracy, which is the empirical correlation between predicted and simulated *DGV *in a validation set. We implemented this fBayesB approach in Fortran F90.

When studying only the main genetic effects via M1, the results of fBayesB are compared with BayesB [4]. A Fortran implementation of BayesB of Berry & Stranden is available on http://www.genomicselection.net (obtained Sep 4, 2009). This version was extended to include dominance effects using a concatenated matrix (***X**_a _**X**_d_*). In principle, it would be possible to additionally consider epistatic effects in BayesB, but this tool would probably require a few months to finish an adequate number of MCMC sampling rounds for a single simulated dataset.

### 2.3 Simulation study

#### Data generation

The simulated population is built up in such a way that it reflects a realistic dairy cattle population. We applied a mutation-drift model and simulated a population with effective population size of 100 animals and 52 273 single nucleotide polymorphisms (SNPs) on a 30 Morgan genome (in style of the Illumina Chip BovineSNP50 and based on Btau4.0 [24]). Details of the genome set-up can be found in Melzer *et al. *(Melzer, Wittenburg, Repsilber: Simulating a more realistic genotype-phenotype map for development of methods to predict phenotypes based on genome-wide marker data - the livestock perspective, submitted). Starting with homozygous loci, a mutation rate of 2.5 · 10^-3 ^per generation was chosen for each SNP locus and 400 generations of random mating involving recombination events on the genome were carried out. About 10% of the loci were fixed due to drift. The LD was measured as *r*^2 ^[25] and the average LD of adjacent SNPs was observed as *r*^2 ^= 0.12. The average SNP heterozygosity was 0.33. The training generations 401 and 402 each consisted of 50 half-sib families with 20 offspring. These individuals were genotyped and phenotyped (*n *= 2 000). The test generations 403 and 404 were built up the same way but without phenotyping the animals. Two main scenarios were set up which differed in the number of QTL. Either 23 or 230 SNP loci were randomly chosen from loci with minor allele frequency (MAF) > 0.02 in generation 400 to be the QTL. Main genetic effects (i.e. additive and dominance effects) were assigned to all QTL. Motivated by the findings of Hayes and Goddard [26], allele substitution effects were drawn from a gamma distribution with shape parameter *α *= 0.42 and scale parameter *β *= 2.619 (23-QTL scenario) or *β *= 8.282 (230-QTL scenario) similar to Meuwissen *et al. *[4]. The sign of an allele substitution effect was drawn at random with equal chance. The degree of dominance was drawn from a normal distribution with mean *m *= 0.193 and variance *τ*^2 ^= 0.312^2 ^[27]. The dominance effect was determined as the product of the absolute allele substitution effect and the degree of dominance. Epistatic effects were included optionally. This means, the genotypic information was used twice: either genotypic values were calculated with main effects only (simulation without epistasis) or genotypic values included main and epistatic effect (simulation with epistasis). For each source of epistasis, six (57) pairs of SNPs were randomly chosen out of the 23 (230) loci to cause interactions. Epistatic effects were drawn from normal distributions with arbitrary parameters chosen such that epistasis explained approximately 25% of the total genetic variance. Different parameters were used for each source of epistatic variation; the parameters are listed in Table 1. To obtain residual error terms, which should be comparable between simulations with and without epistasis, the residual variance component was determined depending on the chosen broad-sense heritability of *H*^2 ^∈ {0.5, 0.3, 0.1}. As an example, *H*^2 ^= 0.5 results in a narrow-sense heritability of *h*^2 ^= 0.474 (*h*^2 ^= 0.307) without (with) simulated epistasis in the 23-QTL scenario. The 23-QTL scenario was repeated 100 times for every *H*^2 ^and the 230-QTL scenario was repeatedly simulated only for *H*^2 ^= 0.5.

**Table 1:** Mean (***m***) and variance (***τ***^**2**^) of normal distributions to simulate epistatic genetic effects

	23-QTL scenario	230-QTL scenario
additive × additive	*m *= 0.2, *τ*^2 ^= 0.3	*m *= 0.02, *τ*^2 ^= 0.03
additive × dominance	*m *= 0.2, *τ*^2 ^= 0.3	*m *= 0.02, *τ*^2 ^= 0.03
dominance × additive	*m *= 0.2, *τ*^2 ^= 0.2	*m *= 0.02, *τ*^2 ^= 0.02
dominance × dominance	*m *= 0.2, *τ*^2 ^= 0.1	*m *= 0.02, *τ*^2 ^= 0.01

#### Scale of genetic effects

For convenience, the phenotypes were simulated on the basis of an F_∞ _model, but the genetic effects were estimated on the orthogonal scale. We employed the equivalence between the representations of genotypic values in (1) and (2) to obtain the translation between scales [22]. With no epistasis simulated, the allele substitution effect *a_j _*and dominance effect *d_j _*were translated into the effects *g_a,j _*and *g_d,j _*on the orthogonal scale by

$$\left(\begin{array}{c} \mu^{*}\\ g_{a,j}\\ g_{d,j} \end{array}\right)\;=\;S_{A,j}^{-1}S\;\left(\begin{array}{c} \mu \\ a_{j}\\ d_{j} \end{array}\right).$$

If epistasis was simulated, the genetic effects on the orthogonal scale were determined for all locus combinations *j *and *k *and the main genetic effects were achieved as the marginal effects. On the F_∞ _scale, we denote the vector of effects ***α***_*j,k *_= (*μ*, *a_j_, d_j_, a_k_, aa_j,k_, da_j,k_, d_k_, ad_j,k_, dd_j,k_*)' and on the orthogonal scale$\alpha_{j,k}^{*}={\left(\mu^{*},\;g_{a,j},\;g_{d,j},\;g_{a,k},\;g_{aa,j,k},\;g_{da,j,k},\;g_{d,k},\;g_{ad,j,k},\;g_{dd,j,k}\right)}^{\prime }$. The translation for a single locus combination was

$$\alpha_{j,k}^{*}\;=\;\left(S_{A,k}^{-1}⊗S_{A,j}^{-1}\right)\left(S⊗S\right)\alpha_{j,k},$$

which directly led to the epistatic effects on the orthogonal scale. Due to the standardisation step in (3), the derived epistatic effect had to be multiplied by the corresponding scaling term. As an example for $ad,\;g_{ad,j,k}\mapsto g_{ad,j,k}\sqrt{2p_{j}\left(1-p_{j}\right)}2p_{k}\left(1-p_{k}\right)$. The derivation of main genetic effects was more difficult. In order to avoid double counting, we considered the main effects separately and collected the contribution of interactions over the genome while the main effects were set to zero (this vector is denoted as ***α***_*j*=0,*k*=0_). The components of interest were obtained from

$$\begin{array}{c} \left(\begin{array}{c} g_{a,j}\\ g_{d,j} \end{array}\right)\;=\;{\left[S_{A,j}^{-1}S\;\left(\begin{array}{c} \mu \\ a_{j}\\ d_{j} \end{array}\right)\;\right]}_{2,3}\\ +{\left[\overset{m}{\underset{k=1,j<k}{\sum }}\left(S_{A,k}^{-1}⊗S_{A,j}^{-1}\right)\left(S⊗S\right)\alpha_{j=0,k=0}\right]}_{2,3}\\ +{\left[\overset{m}{\underset{k=1,j>k}{\sum }}\left(S_{A,j}^{-1}⊗S_{A,k}^{-1}\right)\left(S⊗S\right)\alpha_{k=0,j=0}\right]}_{4,7}. \end{array}$$

Note that the order of loci (either *j *<*k *or *k *<*j*) is necessary to assign the contribution of epistasis correctly to the different sources of genetic variation. Again, each main genetic effect was multiplied by the relevant standard deviation term.

#### Hyper-parameters and other settings

The parameter *λ_s_*, which reflects the prior uncertainty about a genetic effect, was determined indirectly through the choice of the total prior variance. For *s *∈ {*a, d, aa, ad, da, dd*}, we assume that

(4) $$\begin{array}{c} 1=\overset{m_{s}}{\underset{j=1}{\sum }}\text{Var}\left(g_{s,j}\right)=m_{s}\gamma_{s}\frac{2}{\lambda_{s}^{2}}\\ \Rightarrow \lambda_{s}\;=\;\sqrt{2m_{s}\gamma_{s}}. \end{array}$$

In this study, we involved prior knowledge about the proportion of non-zero effects of the genetic variation source *s *in the simulated dataset and chose *γ_s _*accordingly. In the 23-QTL scenario we set *γ_a _*= *γ_d _*= 0.005 and *γ_s _*= 10^-6 ^for *s *∈ {*aa, ad, da, dd*}. In the 230-QTL scenario we applied *γ_a _*= *γ_d _*= 0.05 and *γ_s _*= 10^-6 ^for the epistatic effects. We will return to the issue of parameter choice in the Section Discussion.

Furthermore, to limit the number of iterations, we chose *k*_max _= 1 000 and for the convergence criterion we used *L *= 10^-8 ^for M1. Owing to the computational effort we set *k*_max _= 200 and *L *= 10^-6 ^for M2. Results are reported only for those repetitions where convergence was achieved.

In BayesB the main genetic effects were estimated simultaneously over the whole genome. A hyper-parameter *π *was required to give the proportion of non-zero genetic effects in total; we set π = 0.005 (π = 0.05) in the 23-QTL (230-QTL) scenario. Furthermore, we carried out 50 000 MCMC iterations (40% were neglected as burn-in) and within each iteration 1 000 rounds of the Metropolis-Hastings algorithm were employed.

#### Outline of data analysis

To begin with, we used every 10-th marker (*m *= 5 227), which included the true positions of the simulated QTL, in the statistical analysis. With this reduced genotype dataset, we evaluated differences in parameter estimation between fBayesB and BayesB based on the model with additive and dominance effects. A main issue was to study the impact of including or not including pairwise epistatic effects on the accuracy of genetic value prediction with fBayesB. The influence of a varying proportion of genetic variation on the accuracy of prediction was obtained by analysing the data produced with different broad-sense heritabilities. Further, we studied the consequences of spreading the genetic variation over a multitude of loci with almost equal amounts of variation in each source of genetic variation. In a next step, we used all SNP information (*m *= 52 273) without pre-selection of loci for the estimation of genetic effects and explored the applicability of fBayesB for a large genotype dataset. Finally, to study practical suitability, we estimated genetic effects in a sample of a heterogeneous stock of mice. Genotype and phenotype data are publicly available at http://gscan.well.ox.ac.uk/[28].

## 3 Results

On average 567 loci per dataset had MAF ≤ 0.01. These loci were omitted, but loci deviating from HWE (on average one locus per dataset) were not excluded from the analysis. The average LD between adjacent SNPs was *r*^2 ^= 0.07 in the reduced genotype dataset with 5 227 SNPs.

The differences between fBayesB and BayesB on the basis of M1 are compared. Table 2 shows the average estimated variance components and the average correlation between predicted and simulated genetic values in the 23-QTL scenario. The accuracy between the methods differed only slightly, *ρ *= 0.98 when no epistasis was simulated and *ρ *= 0.78 with simulated epistasis. Both in simulations with and without epistasis, the estimated variance components were similarly biased with BayesB and fBayesB, i.e., the relative bias of the estimate ${\hat{\sigma }}_{a}^{2}$ was -2% (-7%) and the relative bias of ${\hat{\sigma }}_{d}^{2}$ was -13% (-26 to -27%) without (with) simulated epistasis. Though fBayesB only required a small fraction of computing time compared to BayesB (one second versus about six hours on a 2.93 GHz multi-user system), there was neither a lack of accuracy nor differences in bias of variance component estimation.

**Table 2:** Average estimated variance components (standard deviation in brackets) and average accuracy *ρ *of genetic value prediction*

Simulation without epistasis									
Method	Model	$\sigma_{a}^{2}$	$\sigma_{d}^{2}$	$\sigma_{aa}^{2}$	$\sigma_{ad}^{2}$	$\sigma_{da}^{2}$	$\sigma_{dd}^{2}$	$\sigma_{e}^{2}$	*ρ*
BayesB	M1	0.743	0.035	-	-	-	-	0.775	0.980
		(0.578)	(0.039)					(0.605)	
fBayesB	M1	0.742	0.035	-	-	-	-	0.752	0.978
		(0.579)	(0.039)					(0.587)	
fBayesB	M2	0.748	0.039	0.008	0.007	0.007	0.008	0.638	0.959
		(0.583)	(0.041)	(0.013)	(0.016)	(0.014)	(0.017)	(0.484)	
*Simulated components*		*0.757*	*0.040*	-	-	-	-	*0.798*	-

Simulation with epistasis									
Method	Model	$\sigma_{a}^{2}$	$\sigma_{d}^{2}$	$\sigma_{aa}^{2}$	$\sigma_{ad}^{2}$	$\sigma_{da}^{2}$	$\sigma_{dd}^{2}$	$\sigma_{e}^{2}$	***ρ***

BayesB	M1	1.313	0.158	-	-	-	-	2.721	0.785
		(0.681)	(0.131)					(0.874)	
fBayesB	M1	1.310	0.161	-	-	-	-	2.619	0.781
		(0.687)	(0.132)					(0.845)	
fBayesB	M2	1.338	0.193	0.299	0.138	0.065	0.057	1.811	0.833
		(0.688)	(0.142)	(0.215)	(0.111)	(0.071)	(0.070)	(0.598)	
*Simulated components*		*1.409*	*0.217*	*0.346*	*0.133*	*0.089*	*0.020*	*2.214*	-

*23-QTL scenario with 5 227 markers and *H*^2 ^= 0.5. Variance components for each source of genetic variation: $\sigma_{a}^{2}$ additive genetic, $\sigma_{d}^{2}$ dominance, $\sigma_{aa}^{2}$ additive × additive, $\sigma_{ad}^{2}$ additive × dominance, $\sigma_{da}^{2}$ dominance × additive, $\sigma_{dd}^{2}$ dominance *× *dominance; residual variance $\sigma_{e}^{2}$. M1 includes additive and dominance effects, M2 includes additive, dominance and pairwise epistatic effects.

**Figure 1:**
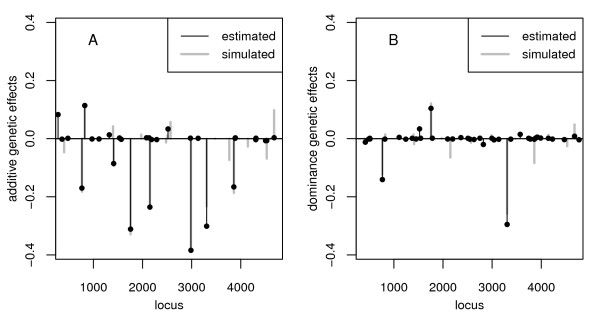
**Estimates of genetic effects if epistasis was absent in the 23-QTL scenario**. (A) Additive and (B) dominance effects for a single dataset via M1 using fBayesB. Filled circles were plotted for each estimated effect > 10^-4^. Single accuracy of genetic value prediction was 0.946.

The additive and dominance effects were estimated equally well with both BayesB and fBayesB. As an example, Figure 1 displays results for the analysis of a single dataset via fBayesB. It shows that the size and location of large to intermediate additive effects were estimated precisely and the pivotal dominance effects were identified closely. In general, there were nearly no differences in the size of estimates of rather large effects and their position between BayesB and fBayesB. It was observed that via BayesB a lot of tiny (but with an effect size > 10^-4^) genetic effects were estimated over the whole genome, whereas fBayesB concentrated on the large loci.

M1 and M2 results are compared to study the impact of including or not including pairwise epistatic effects on the accuracy of predicting the genetic values in the test generations. As an example, Additional file 1 shows the estimated main genetic and epistatic effects for a single dataset when main and epistatic effects were simulated and modelled jointly. The size and position of large main or large epistatic effects were estimated quite well (visual inspection). Small effects, especially concerning dominance, were neither estimated with correct size nor at the simulated position. When epistasis was simulated in the 23-QTL scenario, we obtained an accuracy of 0.781 with M1, which was 5% less than the accuracy based on the correct model M2 for this application, see Table 2. Furthermore, the genetic variance components were underestimated to a larger extent with M1 than with M2. The relative bias of ${\hat{\sigma }}_{a}^{2}$ and ${\hat{\sigma }}_{d}^{2}$ was -7% and -26%, respectively, based on M1 and -5% and -11%, respectively, based on the correct model M2. In the reverse case, when epistasis was modelled and not simulated, the accuracy was 0.959. Hence the loss of accuracy of genetic value prediction was only 2% when the incorrect model M2 was applied. The relative bias of ${\hat{\sigma }}_{a}^{2}$ and ${\hat{\sigma }}_{d}^{2}$ was -1% and -3%, respectively, based on the incorrect model M2 compared to -2% and -13%, respectively, based on the correct model M1. Thus, even with additionally modelled (nuisance) genetic effects in M2, the bias of variance component estimates did not increase for additive and dominance effects. In conclusion, and as expected, we obtained the best estimates of genetic variance components and the highest possible accuracy in the validation set, when M1 was applied in simulations without epistasis and M2 was used under simulated epistasis, i.e., prediction was done with the true model. The loss of accuracy was, however, low when the incorrect model was applied. The relative proportion of genetic variation that could be assigned to the variation of additive effects was estimated best if the correct model was applied. As an example, in the 23-QTL scenario with simulated epistasis, the true ratio of additive to total genetic variance was 0.613. The estimated ratio was 0.626 based on M2 but 0.884 based on M1, see Table 3.

**Table 3:** Average ratio of additive genetic variance to total genetic variance*

	Simulation without epistasis	
Model	23-QTL scenario	230-QTL scenario
M1	0.953	0.810
M2	0.918	0.581
*Simulated ratio*	0.948	0.945

	Simulation with epistasis	
Model	23-QTL scenario	230-QTL scenario

M1	0.884	0.773
M2	0.626	0.401
*Simulated ratio*	0.613	0.648

*fBayesB was used in both QTL scenarios with 5 227 markers and *H*^2 ^= 0.5. M1 includes additive and dominance effects, M2 includes additive, dominance and pairwise epistatic effects.

The results obtained so far are based on *H*^2 ^= 0.5. The influence of a varying proportion of genetic variation in terms of the broad-sense heritability *H*^2 ^on the accuracy of prediction was investigated. Table 4 displays the decreasing accuracy with decreasing *H*^2^. Simulations with *H*^2 ^= 0.3 and *H*^2 ^= 0.5 yielded similar accuracies with M1; accuracy differed about 3 - 5%. With M2 the differences in accuracy were 6 - 12%. With *H*^2 ^= 0.1 the differences decreased further about 11 - 38%. If the proportion of the genetic variation was 0.1, fBayesB had numerical problems with M2 under the given choice of hyper-parameters; the algorithm converged to a final solution only in 40% of the repetitions (90% for *H*^2 ^= 0.3, 99.5% for *H*^2 ^= 0.5). In repetitions that did not converge (*H*^2 ^= 0.1: 3.5%, *H*^2 ^= 0.3: 0.5%, *H*^2 ^= 0.5: 0%) a fluctuating convergence criterion was observed. In all other cases, the algorithm collapsed for no obvious reason.

**Table 4:** Average accuracy of genetic value prediction depending on broad-sense heritability *H*^2^*

Simulation without epistasis				
Model	*H*^2 ^= 0.5	*H*^2 ^= 0.3	*H*^2 ^= 0.1	*H*^2 ^= 0.5 best 10%
M0	0.958	0.940	0.859	0.786
M1	0.978	0.953	0.844	0.774
M2	0.959	0.897	0.640	0.748

Simulation with epistasis				
Model	*H*^2 ^= 0.5	*H*^2 ^= 0.3	*H*^2 ^= 0.1	*H*^2 ^= 0.5 best 10%

M0	0.741	0.707	0.581	0.618
M1	0.781	0.736	0.582	0.621
M2	0.833	0.718	0.339	0.598

*fBayesB was used in the 23-QTL scenario with 5 227 markers. M0 includes only additive genetic effects, M1 includes additive and dominance effects, M2 includes additive, dominance and pairwise epistatic effects. In case of "best 10%" the accuracy of additive genetic value prediction was determined based on 10% animals with best predicted additive genetic value.

In order to prove that we benefit from additionally modelling non-additive genetic effects if those were simulated, we compared the accuracy of genetic value prediction based on M1 with accuracy obtained from a conventional model including only additive genetic effects, called M0. Except for constellations with *H*^2 ^= 0.1, accuracy of M1 was 1-2% (3-4%) higher in simulations without (with) epistasis than accuracy of M0, see Table 4. If we looked at the 10% animals with best predicted additive genetic value (i.e. the breeding value) in simulations with epistasis and *H*^2 ^= 0.5, the accuracy of additive genetic value prediction was 0.618 with M0, 0.621 with M1 and 0.598 with the correct model M2. If we look at the 10% best animals when epistasis was not simulated, the accuracy of additive genetic value prediction was 0.786 based on M0, 0.774 with the correct model M1 and 0.748 with M2. Thus, model choice had an impact on predicting the total genetic values, but if only the extreme breeding values were of interest, e.g. for selection purposes, prediction with a conventional model (M0) was more precise than with the corresponding true model.

**Figure 2:**
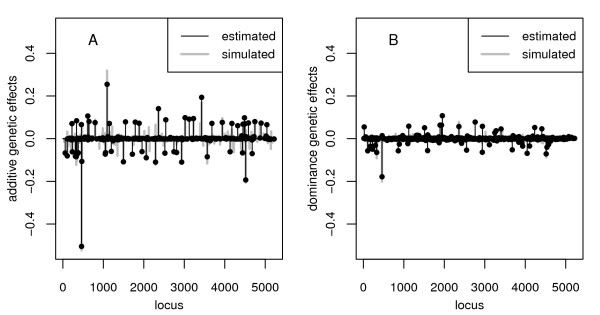
**Estimates of genetic effects if epistasis was absent in the 230-QTL scenario**. (A) Additive and (B) dominance effects for a single dataset via M1 using fBayesB. Filled circles were plotted for each estimated effect > 10^-4^. Single accuracy of genetic value prediction was 0.814.

In a further step, we studied the consequence when the genetic variation was spread over a multitude of loci and compare results obtained with BayesB and fBayesB. Furthermore, the 230-QTL scenario is confronted with the outcomes of fBayesB in the 23-QTL case. When epistasis was not simulated in the 230-QTL scenario, highest accuracy of genetic value prediction was obtained with M1, see Table 5. Though BayesB had a higher relative bias of ${\hat{\sigma }}_{a}^{2}$ (-11% vs. -1%) but crucially smaller bias of ${\hat{\sigma }}_{d}^{2}$ (30% vs. 284%) compared to fBayesB, accuracy was 10% higher. Apparently, dominance has an important impact on genetic value prediction and BayesB could better cope with a larger amount of QTL. fBayesB was able to identify large to intermediate effects, see e.g. Figure 2, but small effects could not be precisely uncovered. BayesB was also superior to fBayesB in terms of accuracy and bias of variance component estimation based on M1 in simulations with epistasis, see Table 5. In any case, fBayesB extremely overestimated variance components of non-additive effects. With application of M1 and fBayesB, the proportion of additive genetic variation to the total genetic variance was underestimated (overestimated) about 13% without (with) simulated epistasis (Table 3). On the basis of M2 this proportion was underestimated by about 25 to 36%.

**Table 5:** Average estimated variance components (standard deviation in brackets) and average accuracy *ρ *of genetic value prediction*

Simulation without epistasis									
Method	Model	$\sigma_{a}^{2}$	$\sigma_{d}^{2}$	$\sigma_{aa}^{2}$	$\sigma_{ad}^{2}$	$\sigma_{da}^{2}$	$\sigma_{dd}^{2}$	$\sigma_{e}^{2}$	*ρ*
BayesB	M1	0.631	0.056	-	-	-	-	0.652	0.860
		(0.204)	(0.035)					(0.180)	
fBayesB	M1	0.699	0.165	-	-	-	-	0.413	0.760
		(0.207)	(0.065)					(0.132)	
fBayesB	M2	0.732	0.304	0.036	0.065	0.068	0.074	0.170	0.608
		(0.214)	(0.112)	(0.028)	(0.036)	(0.042)	(0.046)	(0.066)	
*Simulated components*		*0.709*	*0.043*	-	-	-	-	*0.754*	-

Simulation with epistasis									
Method	Model	$\sigma_{a}^{2}$	$\sigma_{d}^{2}$	$\sigma_{aa}^{2}$	$\sigma_{ad}^{2}$	$\sigma_{da}^{2}$	$\sigma_{dd}^{2}$	$\sigma_{e}^{2}$	*ρ*

BayesB	M1	0.949	0.215	-	-	-	-	1.968	0.585
		(0.250)	(0.067)					(0.266)	
fBayesB	M1	0.920	0.267	-	-	-	-	1.567	0.543
		(0.197)	(0.080)					(0.282)	
fBayesB	M2	1.277	0.910	0.171	0.275	0.296	0.305	0.493	0.340
		(0.230)	(0.269)	(0.086)	(0.106)	(0.127)	(0.126)	(0.257)	
*Simulated components*		*1.284*	*0.192*	*0.308*	*0.103*	*0.071*	*0.014*	*1.952*	-

*230-QTL scenario with 5 227 markers and *H*^2 ^= 0.5. Variance components for each source of genetic variation: $\sigma_{a}^{2}$ additive genetic, $\sigma_{d}^{2}$ dominance, $\sigma_{aa}^{2}$ additive × additive, $\sigma_{ad}^{2}$ additive × dominance, $\sigma_{da}^{2}$ dominance × additive, $\sigma_{dd}^{2}$ dominance *× *dominance, residual variance $\sigma_{e}^{2}$. M1 includes additive and dominance effects, M2 includes additive, dominance and pairwise epistatic effects.

The more QTL were simulated, the less accuracy was observed. If a 10-fold of QTL was responsible for genetic variation, the accuracy of prediction decreased about 22-24% based on M1 and 35-49% based on M2. Since the distances between QTL were smaller than in the 23-QTL scenario, we could expect that LD between loci contributed to the bias of the estimated variance components. For that reason we calculated the empirical variances obtained from the predicted effect-specific genetic values in the validation set, where the epistatic contribution was collected in one component. Table 6 shows that, if only few QTL were given, the missing LD information could be ignored, no matter if epistasis was regarded or not. In contrast, the empirical variance components clearly deviated from those estimated under LE in the 230-QTL scenario, especially if epistasis was modelled. Consequently, the reported variance components in Tables 2, 3 and 5 can only be interpreted as approximations.

**Table 6:** Comparison of empirical variances of predicted genetic values and genetic variance components estimated under LE*

	Simulation without epistasis						
		23-QTL scenario			230-QTL scenario		
Model		$\sigma_{a}^{2}$	$\sigma_{d}^{2}$	$\sigma_{\text{epi}}^{2}$	$\sigma_{a}^{2}$	$\sigma_{d}^{2}$	$\sigma_{\text{epi}}^{2}$

M1	empirical	0.743	0.035	-	0.711	0.163	-
	*under LE*	*0.742*	*0.035*	*-*	*0.699*	*0.165*	*-*
M2	empirical	0.749	0.038	0.030	0.805	0.338	0.278
	*under LE*	*0.748*	*0.039*	*0.030*	*0.732*	*0.304*	*0.243*

	Simulation with epistasis						
		23-QTL scenario			230-QTL scenario		

Model		$\sigma_{a}^{2}$	$\sigma_{d}^{2}$	$\sigma_{\text{epi}}^{2}$	$\sigma_{a}^{2}$	$\sigma_{d}^{2}$	$\sigma_{\text{epi}}^{2}$

M1	empirical	1.309	0.161	-	0.981	0.266	-
	*under LE*	*1.310*	*0.161*	*-*	*0.920*	*0.267*	*-*
M2	empirical	1.332	0.192	0.554	1.442	1.112	1.277
	*under LE*	*1.338*	*0.193*	*0.559*	*1.277*	*0.910*	*1.047*

*fBayesB was used in both QTL scenarios with 5 227 markers and *H*^2 ^= 0.5. Estimates were obtained as empirical variances of effect-specific genetic values predicted in the validation set (rows "empirical") or as genome-wide sum of locus-specific genetic variances which coincides with the assumption of LE (rows "under LE"). Variance components for each source of genetic variation: $\sigma_{a}^{2}$ additive genetic, $\sigma_{d}^{2}$ dominance, $\sigma_{\text{epi}}^{2}$ joint contribution of all epistatic effects. M1 includes additive and dominance effects, M2 includes additive, dominance and pairwise epistatic effects, LE linkage equilibrium.

Next we used the genome-wide SNP information in the statistical analysis (*m *= 52 273). An average of 5 685 loci per dataset were omitted because MAF ≤ 0.01. An average of nine loci deviated from HWE, but these loci were retained. We set *γ_a _*= *γ_d _*= 0.005 for both QTL scenarios. If only main genetic effects were simulated and modelled in the 23-QTL scenario with *H*^2 ^= 0.5, the additive genetic variance component was obtained as ${\hat{\sigma }}_{a}^{2}=0.796\left(se=0.588\right)$, whereas the dominance variance component was extremely overestimated as ${\hat{\sigma }}_{d}^{2}=\;0.\text{493}$ (*se *= 0.354). The accuracy *ρ *= 0.723 was still reasonably high. In the 230-QTL scenario, the accuracy of prediction reduced to *ρ *= 0.513 and ${\hat{\sigma }}_{a}^{2}=\;0.\text{73}0$ (*se *= 0.251), but ${\hat{\sigma }}_{d}^{2}=\;0.\text{729}$ (*se *= 0.308) was not estimated as well as with the reduced SNP set on the basis of M1. Including the pairwise epistatic effects via M2 exceeded practicability. On the basis of 5 227 SNP, more than 13 million effects had to be estimated for each source of epistatic variation and fBayesB required an average of six hours to converge. If 52 273 SNP markers are included, then approximately 1.3 billion effects have to be estimated for each of the four sources of epistasis. Though most markers or pairs of markers have no effect, their estimated genetic effects will be small but not exactly zero. It was not feasible to estimate about 5 billion effects via M2 under proper numerical precision owing to the restricted capacity of memory space. Furthermore, it is questionable how much computing time is required to execute several rounds of iteration. Thus, with 52 273 SNP markers, only M1 was applied to the simulated data with epistasis. This led to a reduced accuracy of *ρ *= 0.611 (*ρ *= 0.380) in the 23-QTL scenario (230-QTL scenario).

Finally, in the real data example, we regarded *m *= 9 441 SNPs which passed the standard quality checks on HWE and MAF. Rarely missing genotypes for these SNPs were imputed via Beagle 3.2 [29]. We studied an immunological phenotype, i.e. percentage of CD8^+ ^cells, and standardised the vector of observations (*n *= 1 521) to avoid numerical problems. A set of covariates was considered similar to Valdar *et al. *[30]: gender, age, family, litter, cage density, experimenter, month and year of experiment. Phenotypes were corrected for the least-squares estimates of these factors in each iteration of the fBayesB algorithm [20]. We set *γ_a _*= γ*_d _*= 0.001 and *γ_s _*= 10^-6 ^for the epistatic effects. Narrow-sense heritability was similarly estimated among the models (M0: *h*^2 ^= 0.294, M1: *h*^2 ^= 0.295, M2: *h*^2 ^= 0.317), which shows robustness of fBayesB in terms of additive genetic variation, see Table 7. Broad-sense heritability increased with growing model complexity (M1: *H*^2 ^= 0.347, M2: *H*^2 ^= 0.448). Figures depicting estimated effect sizes are given in Additional file 2. The largest effects were observed in the MHC region on chromosome 17, which was also reported by Valdar *et al. *[28]. In total, 88% (65%) of the genetic variation was observed around the MHC with M1 (M2). Though additive genetic effect sizes were nearly the same with all models, an additional dominance effect appeared with M2 on chromosome 17. Furthermore, a large epistatic effect occured between chromosomes 1 and 8. Thus, adding epistatic effects to a statistial model may not necessarily improve genetic value predicition, as investigated by Lee *et al. *[7] (see Section Background), but it helps to specify sources of genetic variation and to identify loci that contribute to variation only through interactions.

**Table 7:** Estimated variance components for the real data example*

Model	$\sigma_{a}^{2}$	$\sigma_{d}^{2}$	$\sigma_{aa}^{2}$	$\sigma_{ad}^{2}$	$\sigma_{da}^{2}$	$\sigma_{dd}^{2}$	$\sigma_{e}^{2}$
M0	0.169	-	-	-	-	-	0.405
M1	0.171	0.030	-	-	-	-	0.378
M2	0.174	0.046	0.000	0.000	0.026	0.000	0.303

*Variance components for each source of genetic variation: $\sigma_{a}^{2}$ additive genetic, $\sigma_{d}^{2}$ dominance, $\sigma_{aa}^{2}$ additive × additive, $\sigma_{ad}^{2}$ additive × dominance, $\sigma_{da}^{2}$ dominance *× *additive, $\sigma_{dd}^{2}$ dominance × dominance; residual variance $\sigma_{e}^{2}$. M0 includes only additive genetic effects, M1 includes additive and dominance effects; M2 includes additive, dominance and pairwise epistatic effects.

## 4 Discussion

### 4.1 Hyper-parameters and convergence

When we investigated the influence of a varying proportion of genetic to phenotypic variance on genetic value prediction in the 23-QTL scenario, it was observed that fBayesB did not fulfil the convergence criterion in all situations. In the extreme case with M2 and *H*^2 ^= 0.1, only 40% of all repetitions converged to a proper final solution and it happened that fBayesB simply aborted. (Usually, the algorithm converged after 13-16 iterations with M1 and after 26-28 steps with M2, but up to a 5-fold of iteration steps were necessary in the 230-QTL scenario.) In order to avoid termination, one could tune the "free" hyper-parameter *λ_s_*, which is responsible for the variation of a genetic effect a priori. For convenience, we assumed that the total prior variance was equal to one for each source of genetic variation *s *∈ {*a, d, aa, ad, da, dd*}, see Equation (4). This prior guess depends on the hyper-parameter *γ_s _*which was equal among *s *∈ {*a, d*} and *s *∈ {*aa, ad, da, dd*}. Thus, it seems necessary to specifically adjust *λ_s _*and/or γ*_s _*to each source of genetic variation.

### 4.2 Proportion of non-zero effects

A preliminary study could show that the choice of the hyper-parameter *γ_s _*strongly influenced the accuracy of genetic value prediction and the ability of the fBayesB algorithm to converge (Melzer, Wittenburg, Repsilber: Simulating a more realistic genotype-phenotype map for development of methods to predict phenotypes based on genome-wide marker data - the livestock perspective, submitted). Since the aim of this paper was to investigate the suitability of fBayesB to cope with non-additive effects in general, we simply involved prior knowledge about the proportion of non-zero genetic effects when the hyper-parameter *γ_s _*had to be specified for each genetic variation source *s *∈ {*a, d, aa, ad, da, dd*}. There are several possibilities which allow for a flexible setting of this hyper-parameter. In an intuitive manner, one could determine *γ_s _*via cross-validation as it was done in Melzer *et al*. Another encouraging approach was presented by Shepherd *et al. *[31], therein called emBayesB. It is a BayesB-like estimation of SNP effects without the time consuming Metropolis-Hastings algorithm, but with an EM algorithm for the estimation of *γ_s_*. It employs a binary variable indicating whether a marker is in LD with a QTL (i.e., it is a non-zero effect). So far, this approach was verified for additive genetic effects via simulations, but it is certainly applicable to the non-additive case as well. Not only the specification of the proportion of non-zero effects in the prior setting, however, is important. This study additionally showed that the more loci were responsible for genetic variation, the worse the genetic parameters were estimated, even though we accounted for this proportion in *γ_s_*. With higher proportions of small to intermediate genetic effects, the bias of estimation seriously accumulates via fBayesB. One way out is to reduce noise by eliminating zero effects. This objective is discussed in the next section.

### 4.3 Reduction of model dimensionality

SNP density continues to increase; soon whole-genome sequences will be used for statistical analysis [32]. The ability to uncover genetic effects with Bayesian MCMC methods worsens with increasing LD due to redundancy between markers [33]. Thus, in order to deal with the huge amounts of data, it becomes important to select relevant information. Selection is conceivable in two general ways.

In order to keep as many parameters as required in the statistical model, one could apply a filtering procedure. The significance of putative non-zero effects might be determined, for example, via a stochastic variable selection approach (SVS). In the field of genomic selection, which is based only on additive effects, an SVS implementation of Meuwissen and Goddard [34] was applied by Calus *et al. *[35] to simulated data as well as by Verbyla *et al. *[36] to dairy cattle data. In case of additional non-additive effects, SVS was developed and already successfully applied to obesity data in a mouse backcross population [37]. In that work, an upper bound of model dimensionality had to be fixed and indicator variables were involved specifying which main and epistatic effect had to be included in the model. The Bayes factor then gave evidence of putative QTL.

Dimensionality can also be reduced non-parametrically. As an example, a subset of SNPs may be selected via filtering based on entropy information and wrapping using a naive Bayesian classifier [38]. Alternatively, an informative set of SNPs can be identified on the basis of LD between loci, called tagSNP [39]. This strategy would probably also reduce the bias in variance component estimation due to LD, because only one marker represents a certain chromosome segment. A haplotyping strategy based on LD information was applied to SNP data in Australian beef cattle [40] but with limited success. The authors reported that about 30 000 SNP markers (and a large number of phenotypic records) are required for accurate breeding value prediction. Thus, we have to work with some contradiction: more markers for higher accuracy but less markers (or only the best markers) to reduce estimation errors. The best solution is probably obtained, when the models used are better able to distinguish between markers with and without effect. Meuwissen [41] presented other options to reduce a set of SNPs based on LD between loci or relatedness between individuals.

### 4.4 Non-additive effects

This study has shown that the inclusion of dominance effects in genetic value prediction improved accuracy compared to purely additive models (Table 4). We found that the incorporation of dominance effects was less challenging than the inclusion of epistasis, and we have made a robust step towards advancing insight into the genetic architecture. Regardless of whether dominance or epistatic effects are considered, adequate data are required to estimate non-additive effects. This is also true for periodic re-estimation of genetic effects. In contrast to genomic selection, where additive effects may be obtained from average yields of progeny of genotyped parents, genotyped individuals need to have an own phenotype (e.g. cows).

In general, and also confirmed in our investigations, parametric methods have difficulties to identify and to estimate epistatic effects. One reason is that the orthogonal decomposition of genetic effects only lead to proper results under idealised conditions (LE, absence of mutation and selection etc.) which are violated in practice [42]. As reviewed and discussed by Calus [5], non-parametric methods (e.g. [43]) have the potential to outperform parametric approaches if non-additive effects are included. With an application to broiler data [44], it was shown that kernel methods had a better predictive ability than parametric methods when genome-wide markers were used. For thousands of SNPs and millions of interactions, fBayesB is still computationally feasible but it shows an inherent bias of variance component estimation. Alternatively, machine learning techniques may discover hidden patterns of gene interaction without assuming their structure [45].

Once gene interactions are discovered, they may be used for mate allocation in livestock breeding, where individuals are mated to achieve favourable non-additive gene combinations to further increase genetic gain [46]. Apart from breeding applications, improved statistical modelling [41] and our cognitive interest in the formation of complex phenotypes will benefit from knowledge about the distribution of non-additive effects over the genome and their size.

### 4.5 Number of simulated QTL

An increase in the number of QTL was accompanied by a reduction in the quality of fBayesB for genetic value prediction. fBayesB was able to identify only the biggest QTL effects in the simulated scenarios, in which (nearly) the same amount of genetic variation was spread over 23 or 230 QTL. Thus, effect size in the 230-QTL scenario was roughly one-tenth of that in the 23-QTL case. This complicated the identification of genetic effects in general and, in particular, of non-additive effects, which contributed very little to the genetic variance when compared with additive effects. Many tiny effects were estimated with BayesB, even if genetic variation was caused by few QTL with large effects. In both QTL scenarios, accuracy of genetic value prediction was at a high level with BayesB. It may be more realistic to assume that most livestock traits are influenced by many loci and therefore best results can be expected with BayesB.

## 5 Conclusion

This simulation study showed that the fast Bayesian method (fBayesB) is convenient for genetic value prediction. It requires only a fraction of computing time compared to a conventional MCMC approach BayesB and also enables estimating pairwise interactions.

The number of simulated QTL, the proportion of genetic to phenotypic variance as well as the quantity of SNP in statistical analyses influenced accuracy of genetic value prediction and bias of variance component estimation. Both methods obtained similar results when few QTL with additive and dominance effects were simulated; the maximum accuracy was 98%. As expected, best results were obtained on the basis of the true model corresponding to the simulated scenario, but the loss of accuracy due to using the incorrect model was limited to 2-5%. If many QTL were responsible for genetic variation, accuracy decreased about 22-49% with fBayesB compared to the few QTL scenario, depending on the model. Accuracy based on modelling only additive and dominance effects was generally superior to the complex model, no matter if epistasis was simulated or not, and an additional gain of 4-10% accuracy was observed with BayesB. To sum up, existing approaches for genome-wide estimation of additive genetic effects can easily and robustly be extended by dominance effects to improve accuracy of genetic value prediction and to get further insight into the genetic architecture. In this simulation study, the inclusion of dominance was more important than involving all pairwise interactions, which did not improve prediction in general.

## Competing interests

The authors declare that they have no competing interests.

## Authors' contributions

DW implemented the statistical methods, carried out the analysis and wrote the manuscript. NM simulated the datasets and contributed to the data analysis. NR raised the initial question, advised on the research and suggested improvements to the manuscript. All authors have read and approved the final manuscript.

## Supplementary Material

Additional file 1 **The figure shows estimates of genetic effects and location if epistasis was present in the 23-QTL scenario**: (a) additive, (b) dominance, (c) additive × additive and (d) additive × dominance effects for a single dataset with M2 using fBayesB. Filled circles were plotted for each estimated effect *>*10^-4^. Location of (e) additive × additive and (f) additive × dominance epistatic effects. Single accuracy of genetic value prediction was 0.851. Click here for file

Additional file 2 **The fBayesB approach was applied to public data on a heterogeneous stock of mice**. Genetic effects were estimated based on the different models including only additive effects (M0), additive and dominance effects (M1), additive, dominance and pairwise epistatic effects (M2). Click here for file
