# Supplementary material for: Including non-additive genetic effects in Bayesian methods for the prediction of genetic values based on genome-wide markers
Source: BMC Genet. 2011 Aug 25;12:74. doi: 10.1186/1471-2156-12-74 (PMC3748015; doi:10.1186/1471-2156-12-74)
Supplement: Additional file 1 — The figure shows estimates of genetic effects and location if epistasis was present in the 23-QTL scenario: (a) additive, (b) dominance, (c) additive × additive and (d) additive × dominance effects for a single dataset with M2 using fBayesB. Filled circles were plotted for each estimated effect >10-4. Location of (e) additive × additive and (f) additive × dominance epistatic effects. Single accuracy of genetic value prediction was 0.851. [file 1471-2156-12-74-S1.PDF]

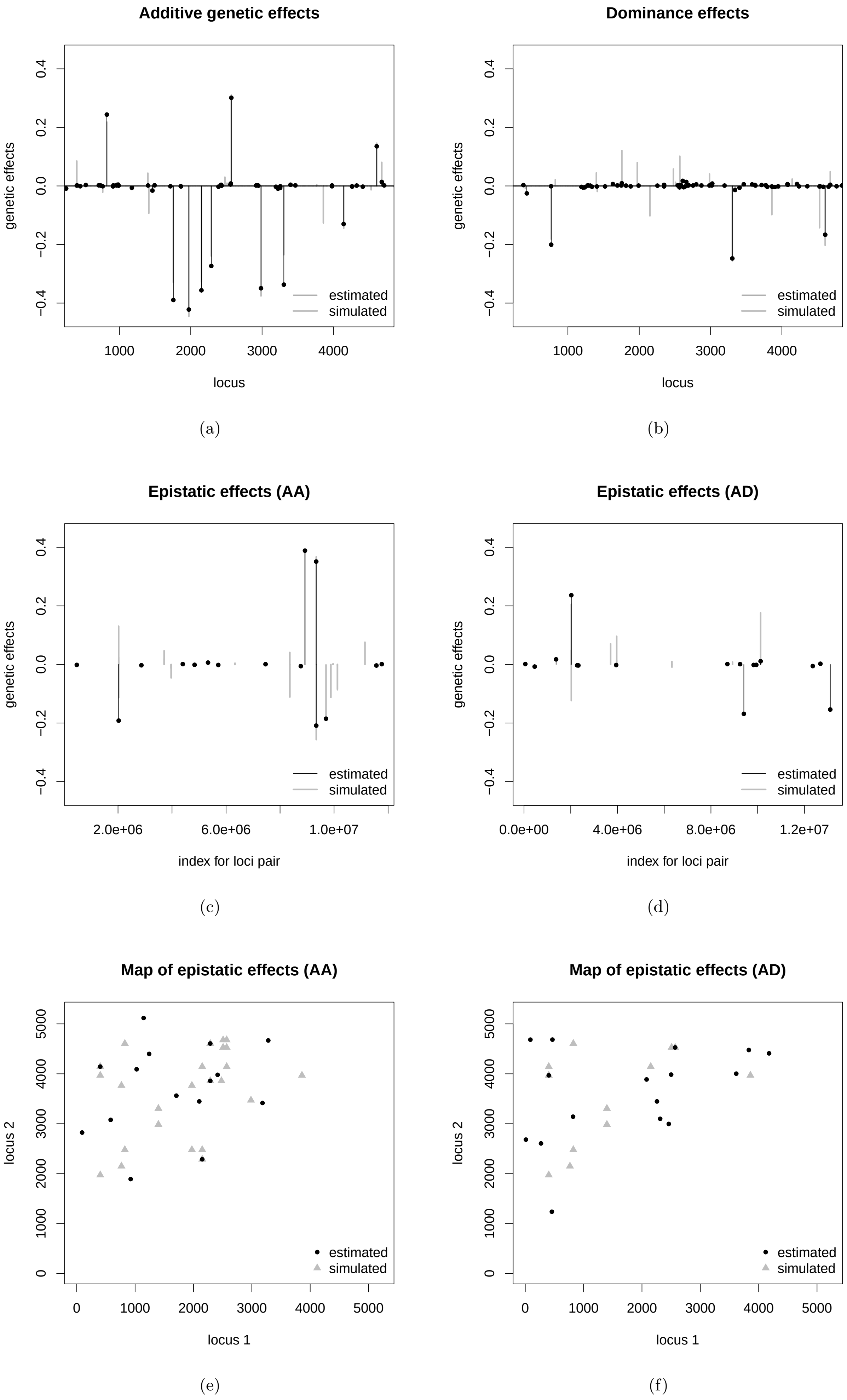

Estimates of genetic effects and location if epistasis was present in the 23-QTL scenario. (a) Additive, (b) dominance, (c) additive $\times$ additive and (d) additive $\times$ dominance effects for a single dataset with M2 using fBayesB. Filled circles were plotted for each estimated effect $> 10^{-4}$. Location of (e) additive $\times$ additive and (f) additive $\times$ dominance epistatic effects. Single accuracy of genetic value prediction was 0.851.
