# Supplementary material for: Including non-additive genetic effects in Bayesian methods for the prediction of genetic values based on genome-wide markers
Source: BMC Genet. 2011 Aug 25;12:74. doi: 10.1186/1471-2156-12-74 (PMC3748015; doi:10.1186/1471-2156-12-74)
Supplement: Additional file 2 — The fBayesB approach was applied to public data on a heterogeneous stock of mice. Genetic effects were estimated based on the different models including only additive effects (M0), additive and dominance effects (M1), additive, dominance and pairwise epistatic effects (M2). [file 1471-2156-12-74-S2.PDF]

1 Model with additive genetic effects (M0)

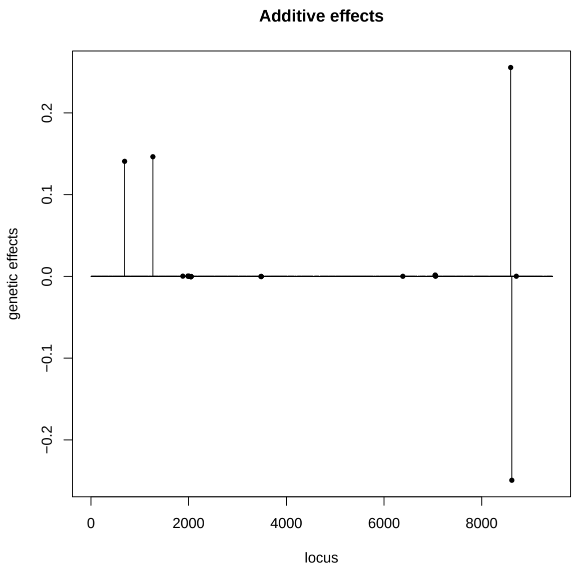

Figure 1: Estimates of additive genetic effects. Filled circles were plotted for each estimated effect $> 10^{-4}$.

2 Model with additive and dominance effects (M1)

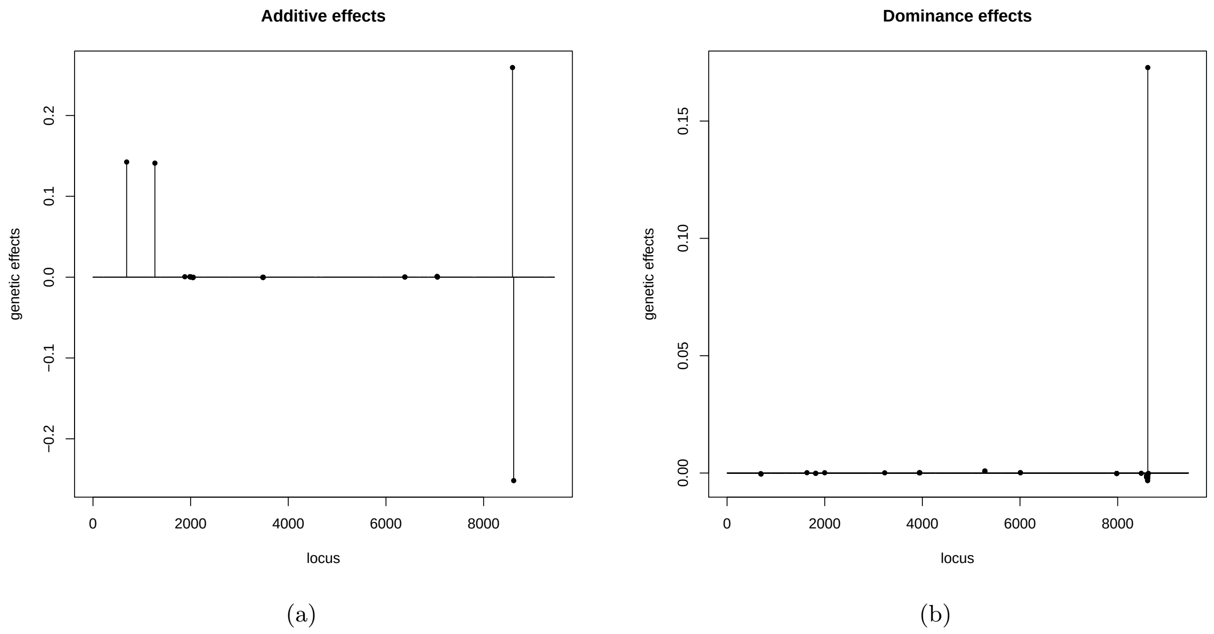

Figure 2: Estimates of main genetic effects. (a) Additive and (b) dominance effects. Filled circles were plotted for each estimated effect $> 10^{-4}$.

3 Model with additive, dominance and epistatic effects (M2)

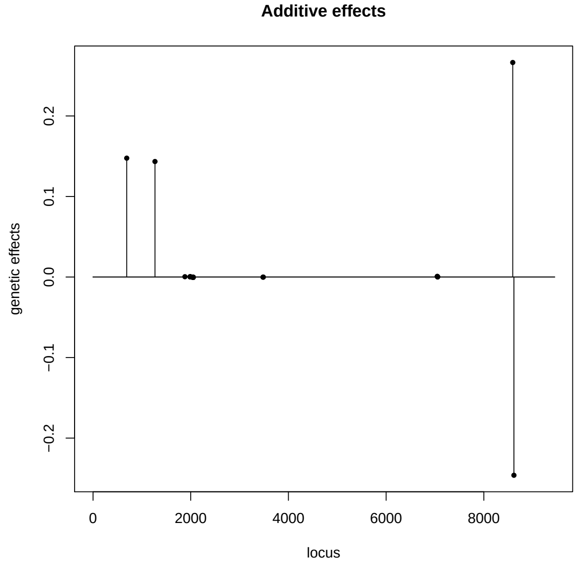

(a)

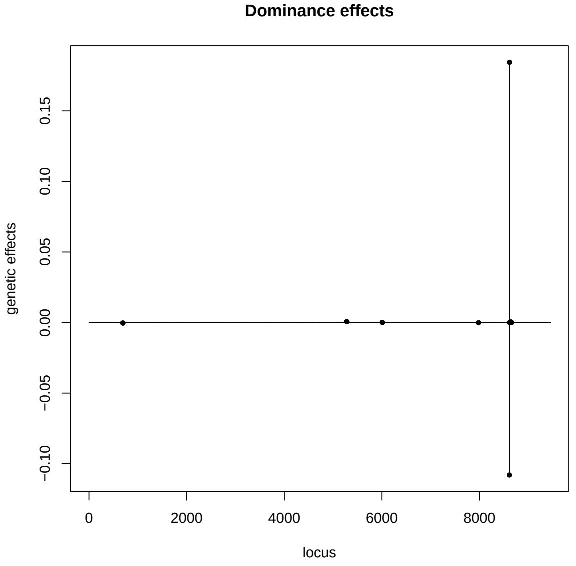

(b)

Figure 3: Estimates of main genetic effects. (a) Additive and (b) dominance effects. Filled circles were plotted for each estimated effect $> 10^{-4}$.

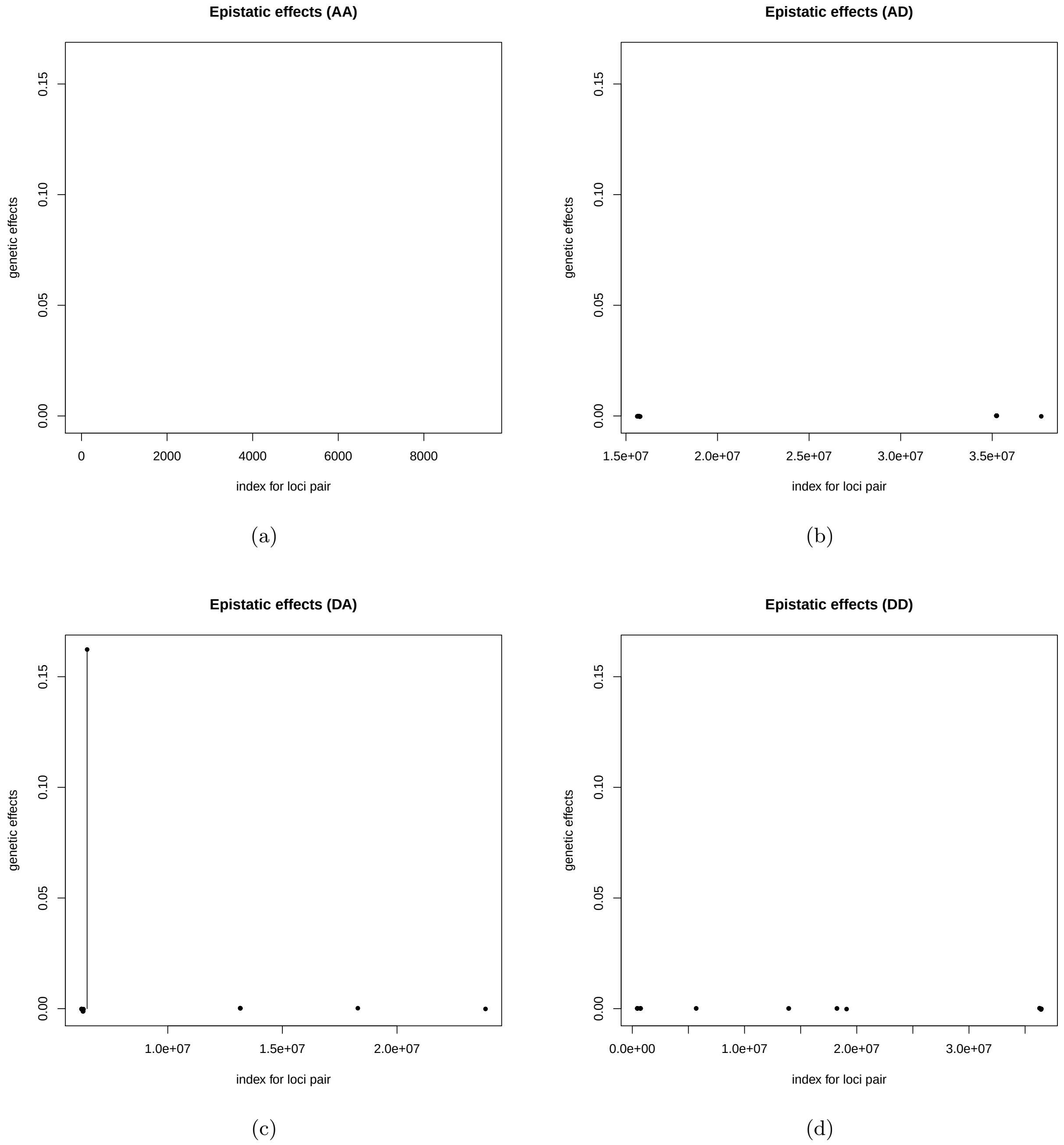

Figure 4: Estimates of epistatic genetic effects. (a) Additive × additive, (b) additive × dominance, (c) dominance × additive and (d) dominance × dominance effects. Filled circles were plotted for each estimated effect $> 10^{-4}$.

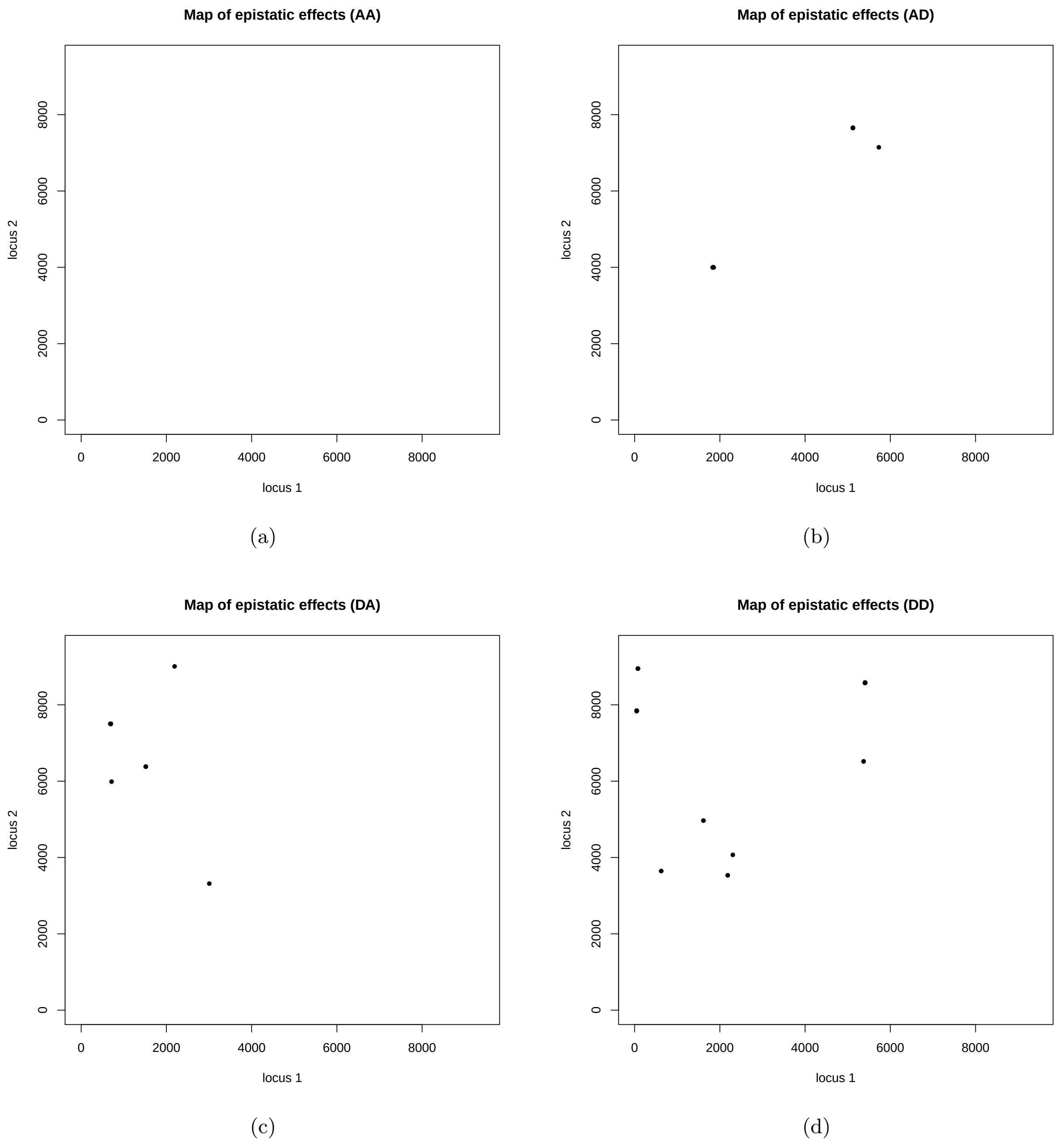

Figure 5: Location of estimated epistatic effects $> 10^{-4}$. (a) Additive × additive, (b) additive × dominance, (c) dominance × additive and (d) dominance × dominance effects.
